# Supplementary figures and images for: Functional geometry of auditory cortical resting state networks derived from intracranial electrophysiology
Source: PLoS Biol. 2023 Aug 31;21(8):e3002239. doi: 10.1371/journal.pbio.3002239 (PMC10499207; doi:10.1371/journal.pbio.3002239)

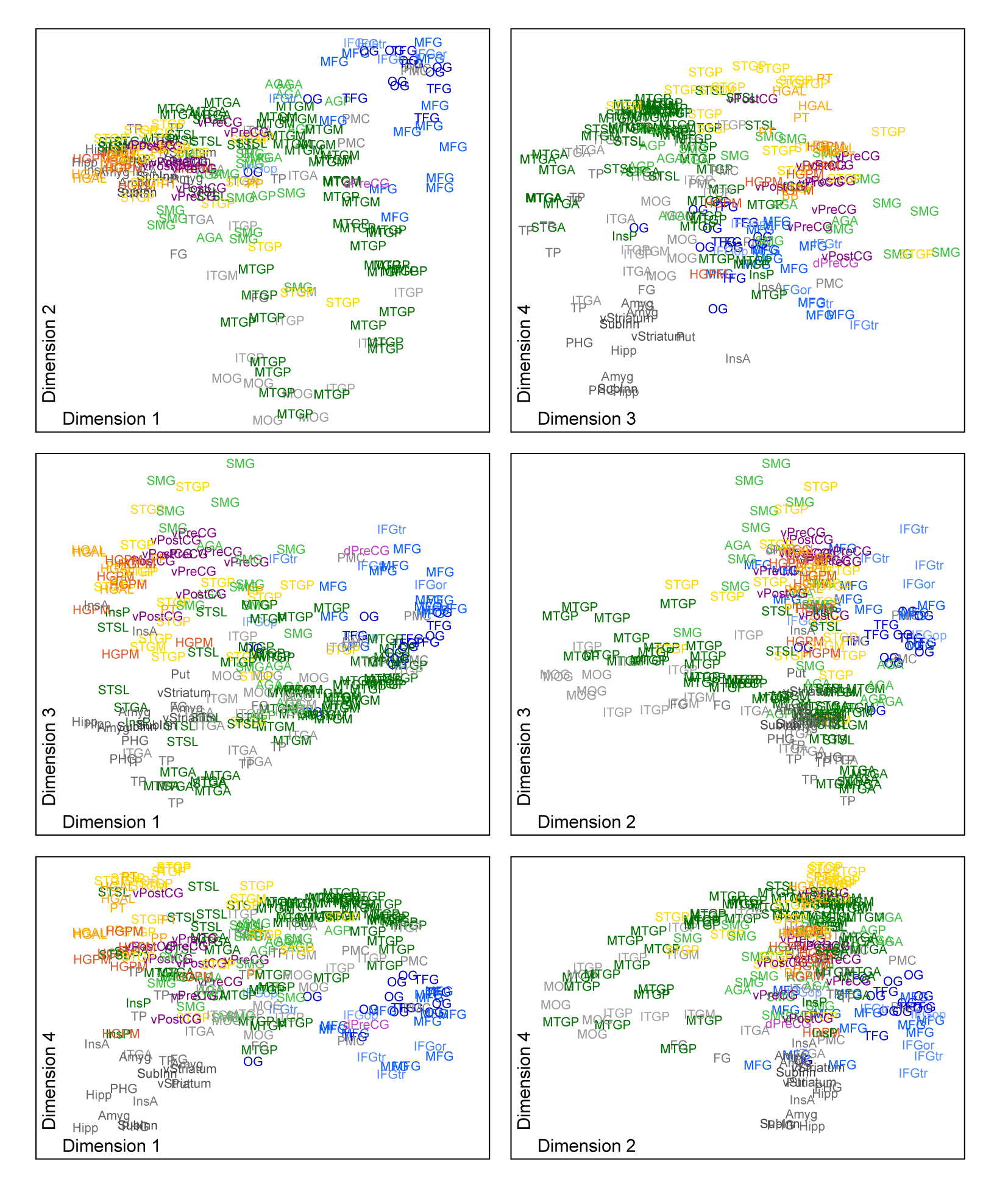

Supplement: S1 Fig — (TIF) [file pbio.3002239.s001.tif]

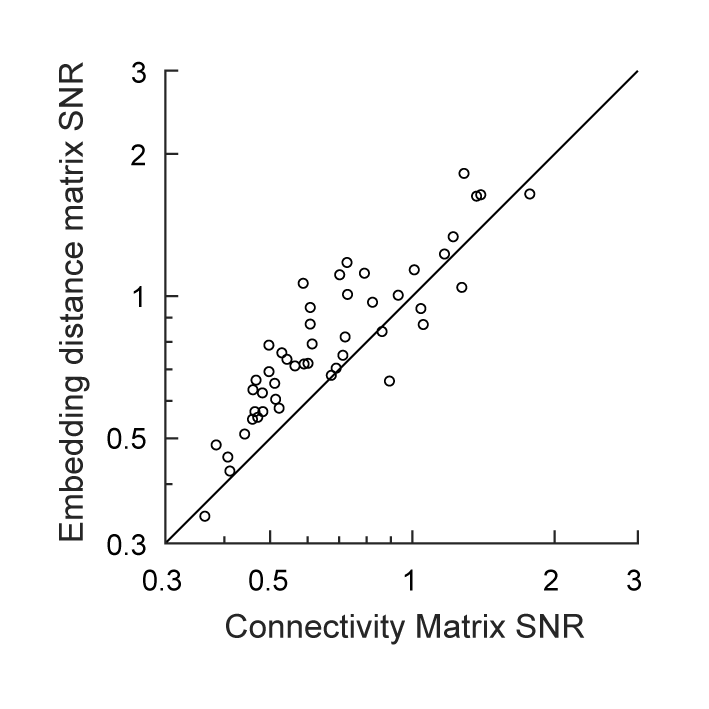

Supplement: S2 Fig — Each symbol corresponds to 1 participant. For each participant, the SNRs of embedding distances and connectivity were calculated from the recorded RS block as described in Methods. In most participants, the embedding analysis exhibited superior SNR characteristics compared to direct analysis of connectivity. (TIF) [file pbio.3002239.s002.tif]

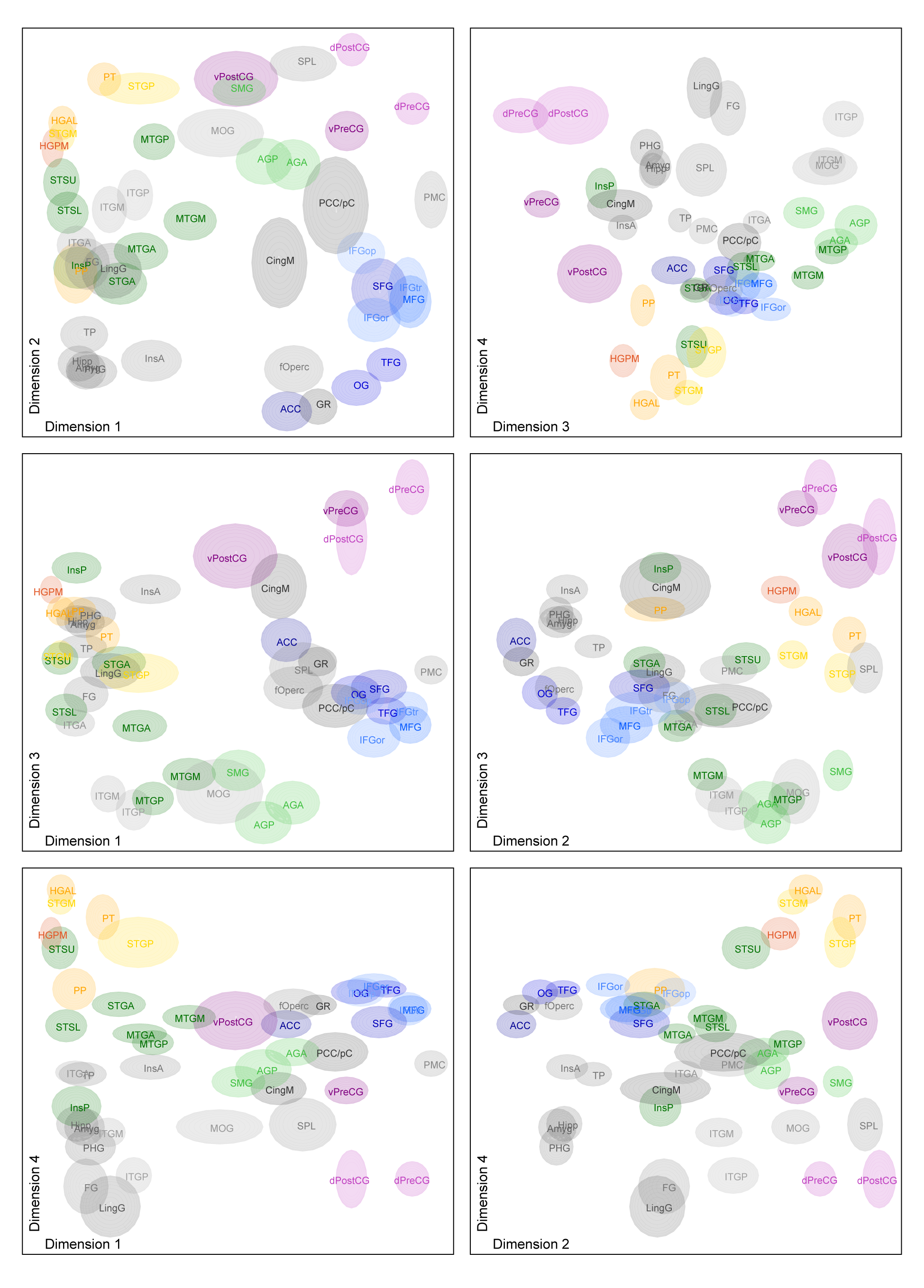

Supplement: S3 Fig — (TIF) [file pbio.3002239.s003.tif]

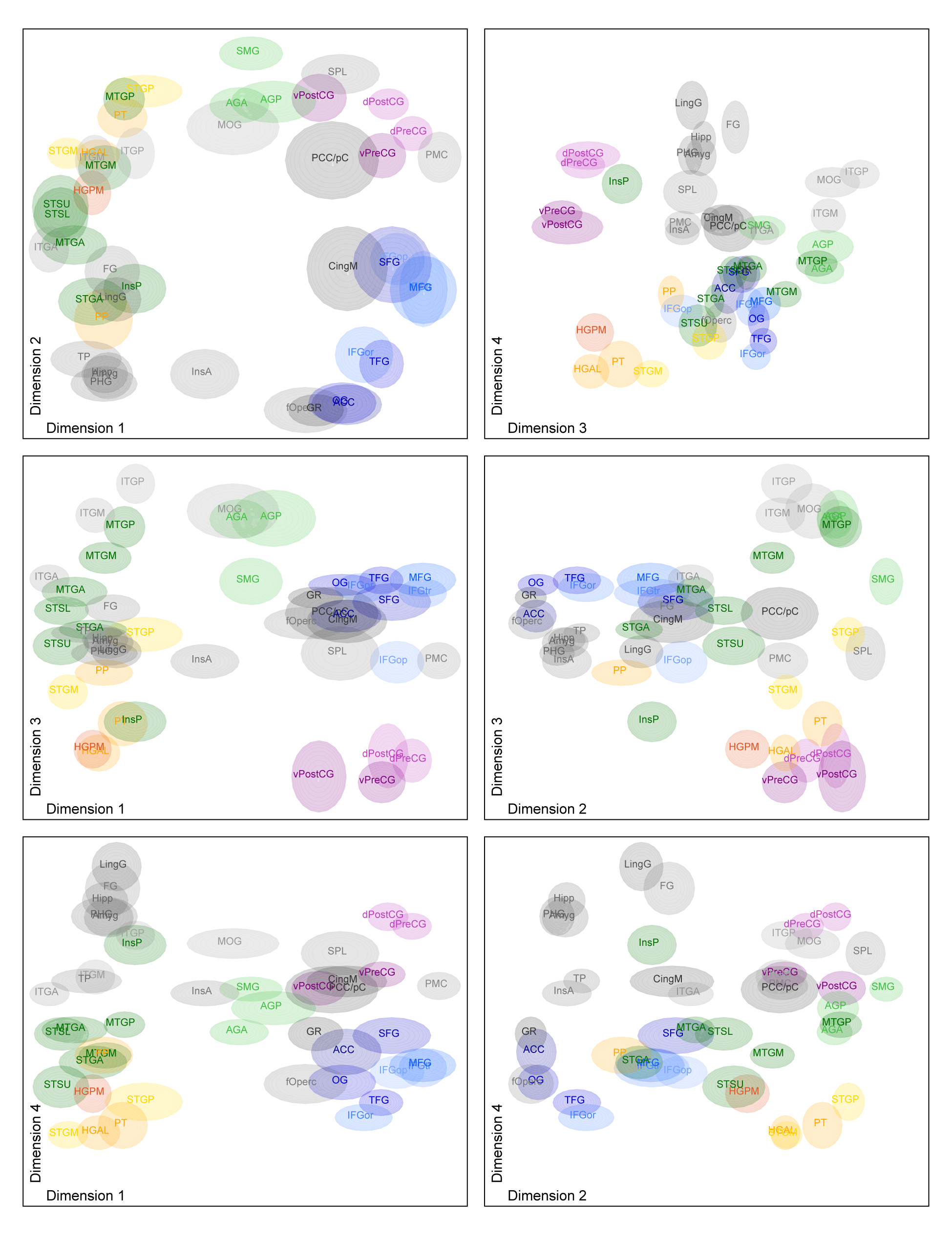

Supplement: S4 Fig — (TIF) [file pbio.3002239.s004.tif]

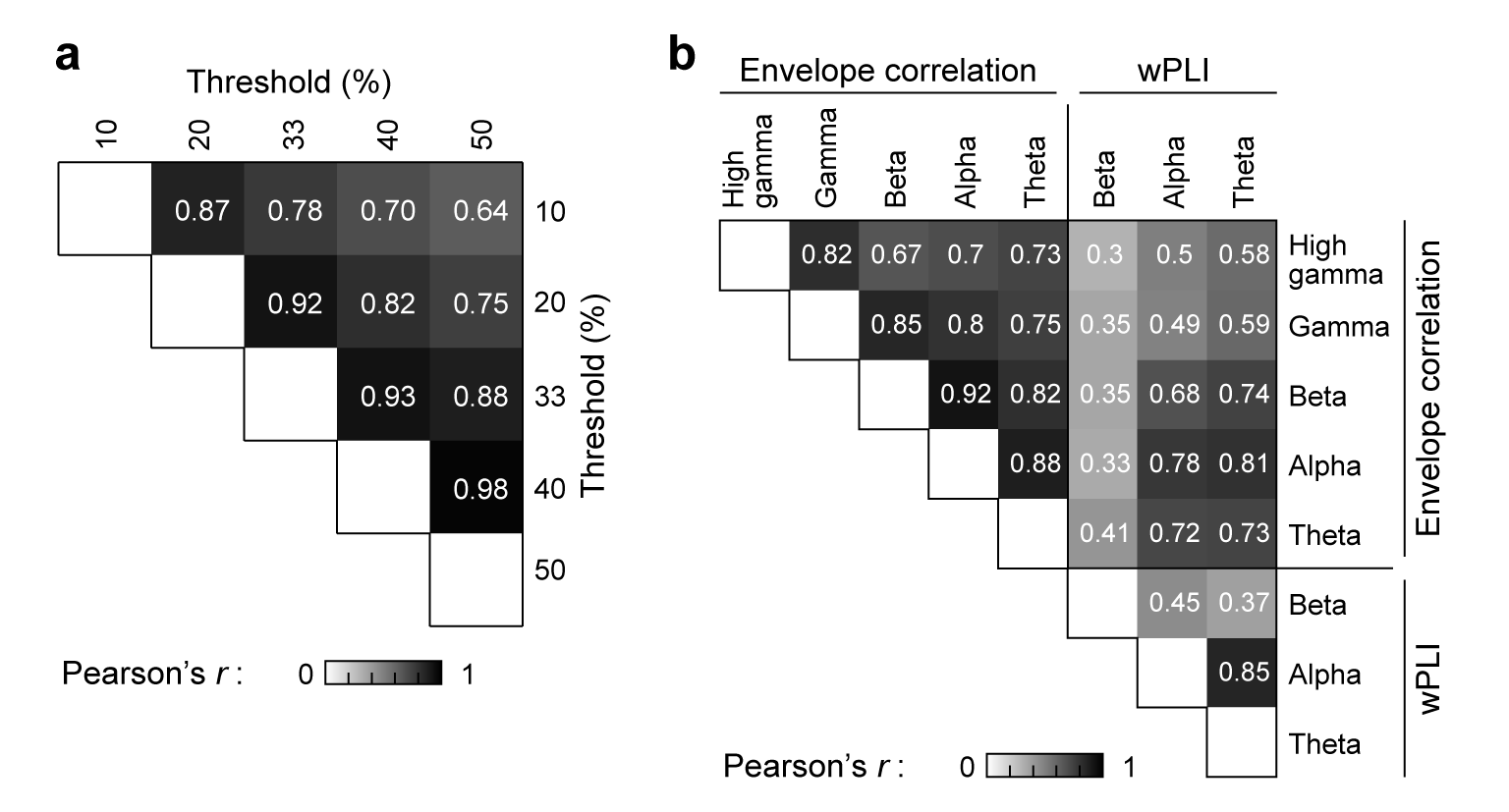

Supplement: S5 Fig — (a) Comparison of embedding results across thresholds applied to connectivity matrices. Threshold = 33% was used for the results of the main figures. (b) Comparison of embedding results across frequency bands and functional connectivity measures. Threshold = 33%. For (a) and (b), data shown are Pearson correlations of inter-ROI distances from embeddings obtained with the different thresholds, measures, and bands. (TIF) [file pbio.3002239.s005.tif]

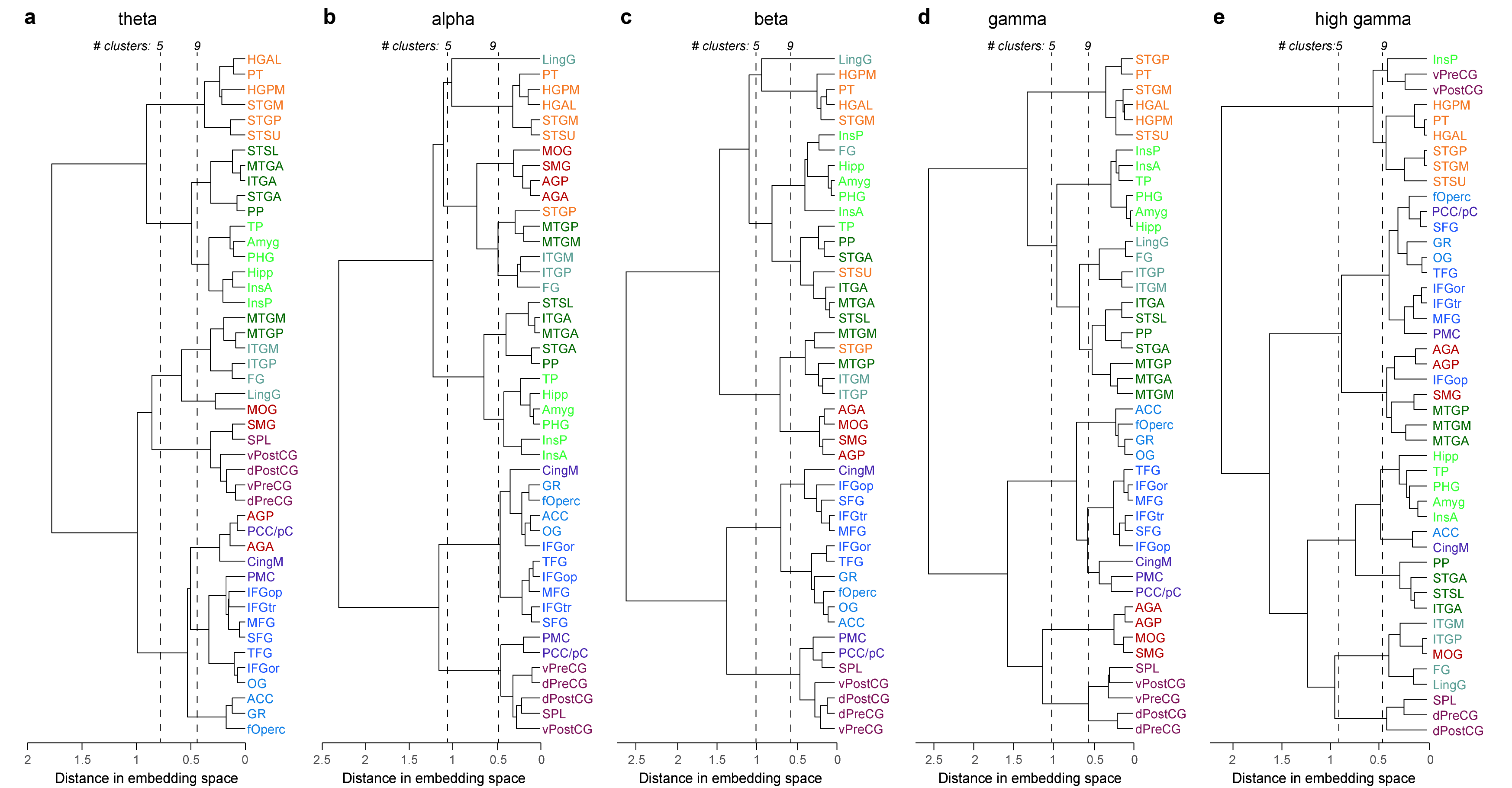

Supplement: S6 Fig — (a) theta; (b) alpha; (c) beta; (d) gamma (same data as in Fig 4); (e) high gamma. Linkages between ROI groups identified using agglomerative clustering. As in Fig 4, 2 thresholds are shown for each band, nCluster = 5 and 9 (vertical dashed lines). The number of clusters is the number of lines in the dendrogram intersected by the threshold line. Clusters consist of all ROIs descending from the intersected line. The color scheme for ROI labels is set by the gamma parcellation. (TIF) [file pbio.3002239.s006.tif]

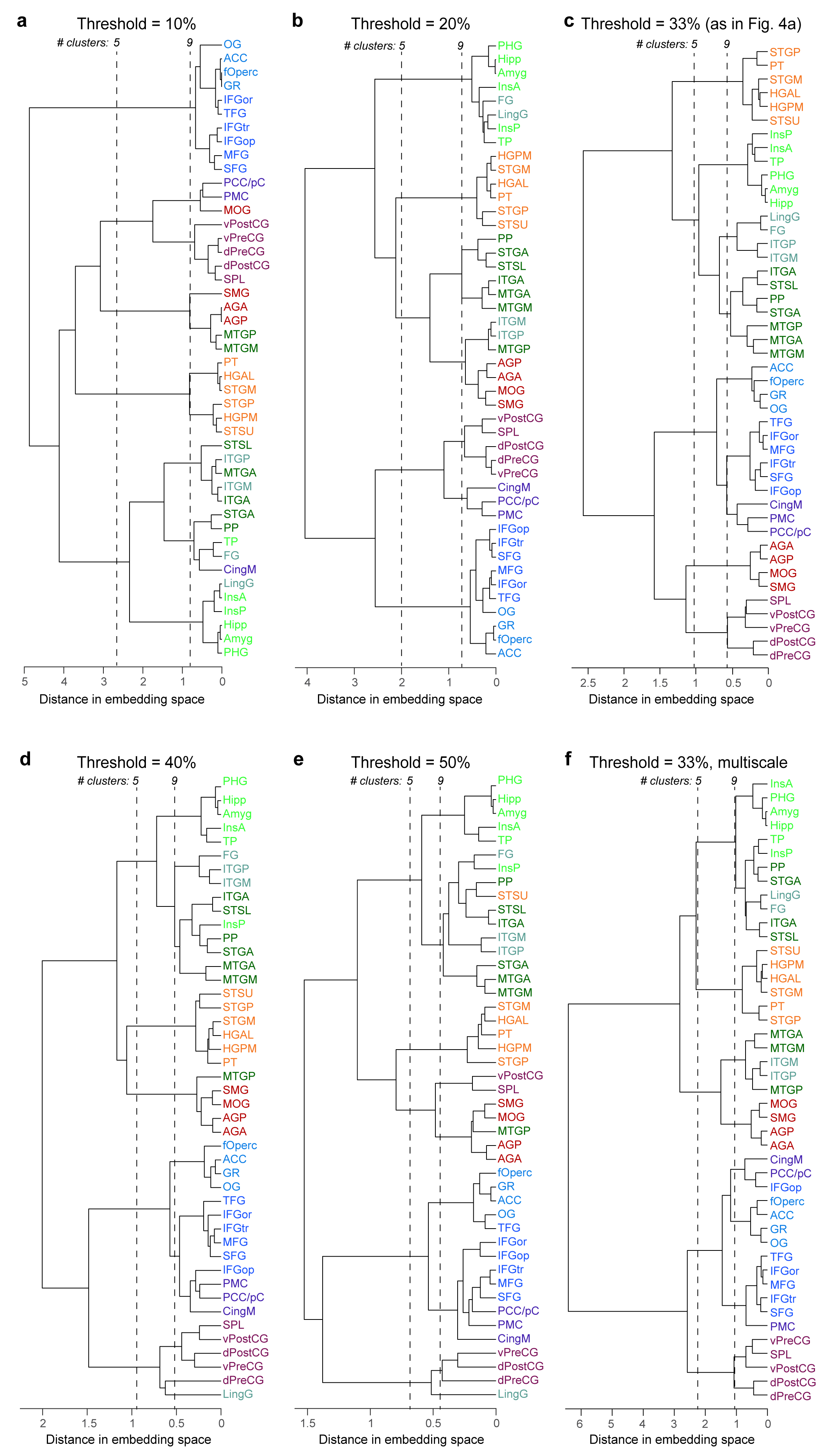

Supplement: S7 Fig — (a-e) Effect of varying threshold from 10%–50% with t = 1. (f) Clustering results for embeddings derived using the multiscale approach instead of t = 1. For all panels, linkages between ROI groups were identified using agglomerative clustering. As in Fig 4, 2 thresholds are shown, nCluster = 5 and 9 (vertical dashed lines). The number of clusters is the number of lines in the dendrogram intersected by the threshold line. Clusters consist of all ROIs descending from the intersected line. The color scheme for ROI labels is set by the gamma parcellation with threshold 33% and t = 1. (TIF) [file pbio.3002239.s007.tif]

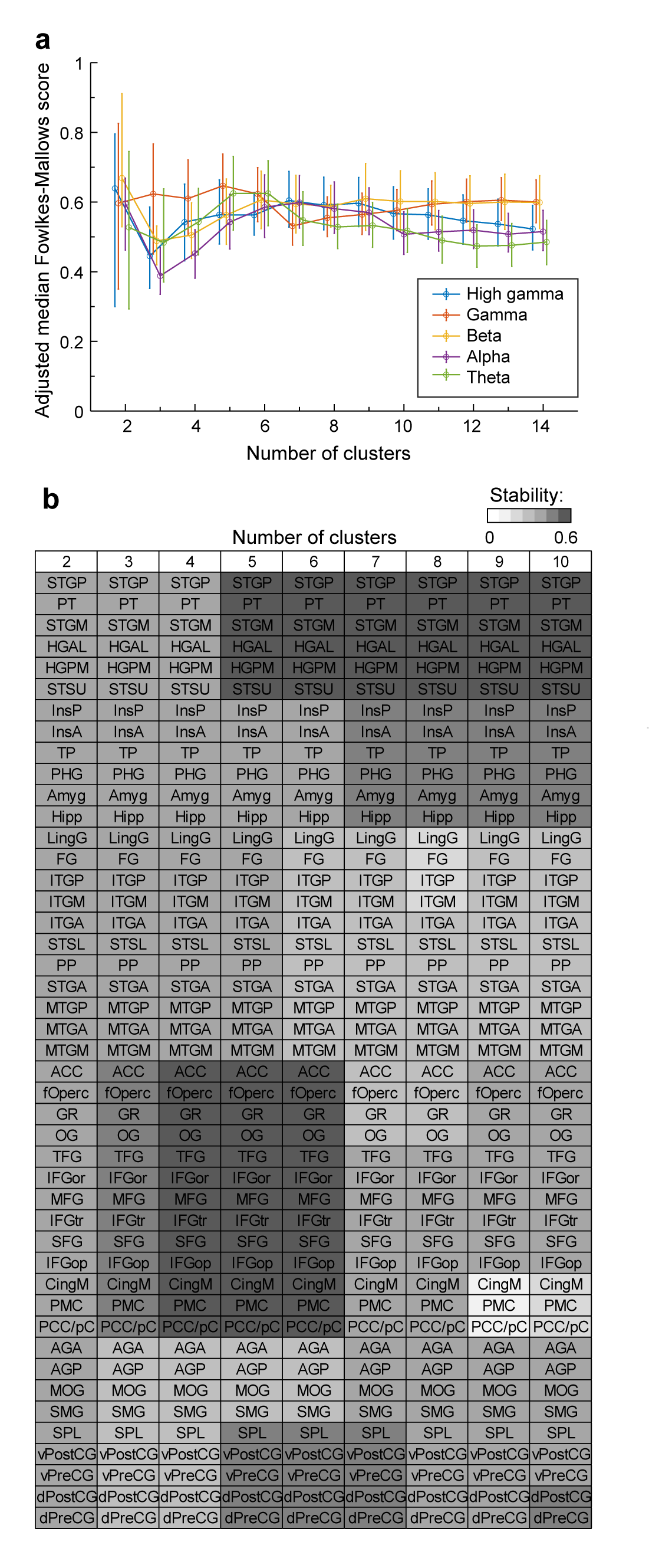

Supplement: S8 Fig — (a) Stability of overall cluster results shown in Fig 4A and S6 Fig was evaluated for each frequency band as a function cluster number using the Fowlkes–Mallows score. (b) Cluster-wise stability for gamma band data as a function of cluster number was evaluated using the Jaccard index. (TIF) [file pbio.3002239.s008.tif]

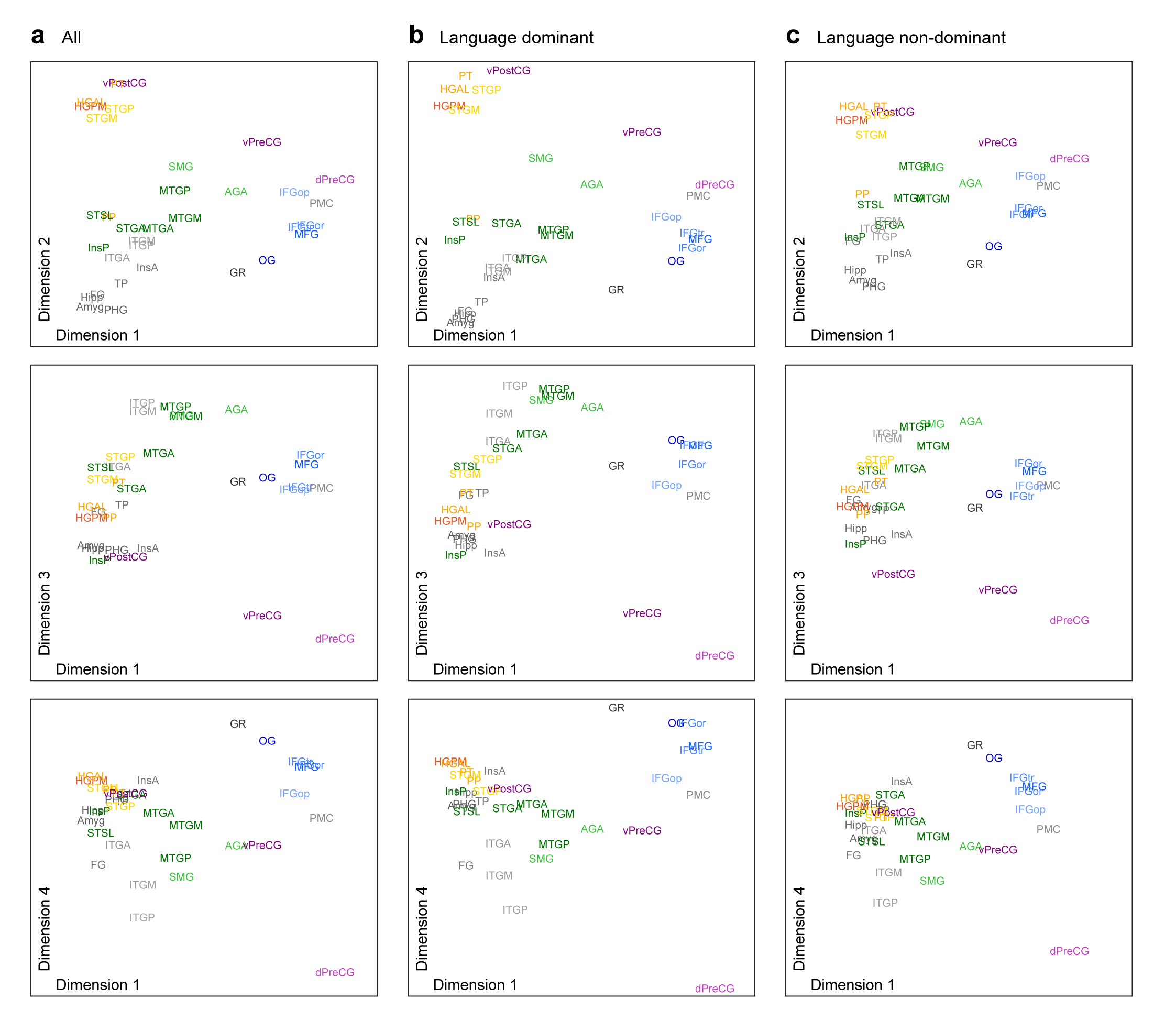

Supplement: S9 Fig — Data plotted on the same scale in the first 4 dimensions of embedding space for all dominant and nondominant participants (a), just dominant (b), and just nondominant (c). (TIF) [file pbio.3002239.s009.tif]
