# Supplementary material for: Functional geometry of auditory cortical resting state networks derived from intracranial electrophysiology
Source: PLoS Biol. 2023 Aug 31;21(8):e3002239. doi: 10.1371/journal.pbio.3002239 (PMC10499207; doi:10.1371/journal.pbio.3002239)
Supplement: S1 Text — (DOCX) [file pbio.3002239.s015.docx]

**S1 Text: Diffusion Map Embedding**

In the framework of DME, we consider a space *X* that is the set of *M* recording sites. We compute the similarity between those sites based on the time varying signals recorded at each site, defining similarity *k*(*x_i_,x_j_*) as the cosine similarity between functional connectivity of nodes *x_i_* and *x_j_*.

Define the matrix **K** whose *i*,*j*^th^ element is *k*(*x_i_,x_j_*). *k*(*x_i_,x_j_*) is required to be symmetric, i.e., *k*(*x_i_,x_j_*) = *k*(*x_j_,x_i_*), and positivity preserving, i.e. *k*(*x_i_,x_j_*) > 0 for all [*i,j*], to allow for spectral analysis of a normalized version of **K**.

From *X* and **K** we can construct a weighted graph Γ in which the vertices are the nodes and the edge weights are *k*(*x_i_,x_j_*). We take random walks on the graph at time steps *t* = 1, 2, …, jumping from node *x_i_* to node *x_j_* at each time step, with the (stochastic) decision as to which node should be visited next depending on *k*(*x_i_,x_j_*).

Define

*p*(*x_i_,x_j_*) = *k*(*x_i_,x_j_*)/*d*(*x_i_*),

where

*d*(*x_i_*) = Σ*_j_*[*k*(*x_i_,x_j_*)]

is the degree of node *x_i_*. Normalizing *k*(*x_i_,x_j_*) in this way allows us to interpret it as the probability *p*(*x_i_,x_j_*) that we'll jump from vertex x_i_ to vertex *x_j_* in a single time step of our random walk.

If we consider a single time step, we only capture the structure in *X* on a very local scale, since we can only jump between vertices that are directly connected. As we run the random walk forward in time, we begin to explore more of our neighborhood, and we begin to explore other neighborhoods as well. Two vertices *x_i_* and *x_j_* that have similar connectivity to the rest of the network have a high probability of being connected during these longer walks because they themselves are connected to similar groups of vertices, and so there are many possible paths between *x_i_* and *x_j_*.

The diffusion operator (matrix) **P** = [*p*(*x_i_,x_j_*)] describes how signals diffuse from node to node in the graph. If **v** is a *M*×1 vector (i.e., a value assigned to each vertex, for example representing an input to each node), then **P** describes what will happen to that input as time goes on.

**Pv** = [*p*(*x*_1_,*x*_1_)**v**[*x*_1_]+*p*(*x*_1_,*x*_2_)**v**[*x*_2_]+…; *p*(*x*_2_,*x*_1_)**v**[*x*_1_]+*p*(*x*_2_,*x*_2_)**v**[*x*_2_]+…;…]^T^

If, for example, all the nodes were insular, with *p*(*x*_i_,*x*_i_)=1 for all *i*, and otherwise *p*(*x_i_*,*x_j_*)=0, **Pv** = **v**, i.e., no diffusion occurs. If the probabilities are more distributed, **Pv** would reveal how much signals diffuse out from each node given the starting condition of **v**. Importantly, **P**^k^**v** would reveal what that distribution looks like after *k* time steps.

The eigenvector expansion of **P** based on its eigenvectors ψ*_j_* and eigenvalues , λ*_j_*, *j* = 1...*M*, is a natural method for uncovering structure in **P** because each eigenvector of **P** is a dimension along which relevant organizational features emerge. That is, clusters of related points (communities) tend to be distinct and ordered along these dimensions. In fact, we could preserve a lot of information about **P** by keeping just a subset of *L* of these vectors and discarding the rest. The information we want to preserve in the context of diffusion map embedding is the functional distance between the data at two nodes given *t* time steps to meander through the graph. We can define the diffusion map

$$\Psi^{(t)}(x_{i})= \left[ {\lambda_{1}}^{t}\psi_{1}\left( x_{i} \right), {\lambda_{2}}^{t}\psi_{2}\left( x_{i} \right), \ldots,{\lambda_{L}}^{t}\psi_{L}\left( x_{i} \right) \right]^{T}$$

which maps each point *x* in *X* to a point in an embedding space of dimension *L* ≤ M. In this space, the diffusion distance *D*, which is the Euclidean distance between points, is the difference in the probability distributions linking *x_i_* to the rest of the network and *x_j_* to the rest of the network:

*D*^(^*^t^*^)^(*x_i_,x_j_*)^2^ = ||Ψ^(^*^t^*^)^(x*_i_*) - Ψ^(^*^t^*^)^(x*_j_*)||*_l_*_2_.= ||*p*^(^*^t^*^)^(*x_i_*,:)- *p*^(^*^t^*^)^(*x_j_*,:)||^2^*_l_*_2_.

To compare embeddings across groups of participants, or modalities of measurements, it is necessary to map embeddings to a common space. To do so, consider two sets of data α and β, and the data spaces *X*_α_ and *X*_β_. The problem is that *X*_α_ and *X*_β_ are different spaces with different kernels *k*_α_ and *k*_β_. This means that the eigenvectors for **P**_α_ and **P**_β_ will be different, and data projected into a space defined by some subset of the eigenvectors cannot be compared directly. The solution is to apply a change of basis operator to one set of the eigenvectors to get the data into the same embedding space [1]:

D^(^*^t^*^)^(*x_i_*_|α_,*x_i_*_|β_) = ||Ψ^(^*^t^*^)^_α_(*x*) − O_β→ α_Ψ^(^*^t^*^)^_β_(*x*)||*_l_*_2_.

Where the change of basis operator O_β→α_ is defined as

O_β→α_ **v** = Σ*_j_*[**v**(*j*)<ψ^(^*^i^*^)^_α_ ,ψ^(^*^j^*^)^_β_>]*_i_*_>=1_,

Where <ψ^(^*^i^*^)^_α_ ,ψ^(^*^j^*^)^_β_> is the inner product of ψ^(^*^i^*^)^_α_ and ψ^(^*^j^*^)^_β_.

A multiscale DME analysis was introduced by Richards and colleagues [2] that considers all scales (i.e., values of *t*) simultaneously, eliminating the need to choose a particular value of this parameter:

$\Psi^{MS}(x_{i})= \left[ \frac{\lambda_{1}}{1-\lambda_{1}}\psi_{1}\left( x_{i} \right), \frac{\lambda_{2}}{1-\lambda_{2}}\psi_{2}\left( x_{i} \right), \ldots, \frac{\lambda_{L}}{1-\lambda_{L}}\psi_{L}\left( x_{i} \right) \right]^{T}$.

**REFERENCES CITED**

1. Coifman RR, Hirn MJ. Diffusion maps for changing data. Applied and Computational Harmonic Analysis. 2014;36(1):79-107. doi: <https://doi.org/10.1016/j.acha.2013.03.001>.

2. Richards JW, Freeman PE, Lee AB, Schafer CM. Accurate parameter estimation for star formation history in galaxies using SDSS spectra. Monthly Notices of the Royal Astronomical Society. 2009;399(2):1044-57. doi: 10.1111/j.1365-2966.2009.15349.x.
