# Supplementary material for: Functional geometry of auditory cortical resting state networks derived from intracranial electrophysiology
Source: PLoS Biol. 2023 Aug 31;21(8):e3002239. doi: 10.1371/journal.pbio.3002239 (PMC10499207; doi:10.1371/journal.pbio.3002239)
Supplement: S2 Text — (DOCX) [file pbio.3002239.s016.docx]

**S2 Text: Fowlkes-Mallows Index**

The content of this appendix uses the functions and notation of Fowlkes and Mallows, 1983 [1] with minor adjustments. Their index, denoted *B_k_*, where *k* is the number of clusters, represents the similarity of clusterings, independent of cluster ordering, as a value between 0 and 1, where 1 represents identical clustering. *B_k_* is given from the following equations:

$$B_{k}=\frac{T_{k}}{\sqrt{P_{k}Q_{k}}}$$

where

$$T_{k}=\sum_{i=1}^{k} \sum_{j=1}^{k} {m_{ij}}^{2}-n$$

$$P_{k}=\sum_{i=1}^{k} \left( \sum_{j=1}^{k} {m_{ij}}^{2} \right)^{2}-n$$

$$Q_{k}=\sum_{j=1}^{k} \left( \sum_{i=1}^{k} {m_{ij}}^{2} \right)^{2}-n$$

…and *M =* [*m_ij_*] is a matrix with *k* rows and *k* columns where each *m_ij_* represents the number of objects in cluster *i* from one clustering and cluster *j* from the other clustering. *n* is the total number of objects clustered. In the case of random assignment to clusters of the sizes observed, however, *B_k_* is biased (not zero), and the expected value for this bias is given by:

$$E\left( B_{k} \right)= \frac{\sqrt{P_{k}Q_{k}}}{n(n-1)}$$

We normalized by this bias value to give a stability index that averages 0 for chance assignment to clusters (with this normalization, values less than 0 are theoretically possible) and 1 for perfect concordance:

$$stability= \frac{B_{k}-E(B_{k})}{1-E(B_{k})}$$

**REFERENCES CITED**

1. Fowlkes EB, Mallows CL. A Method for Comparing Two Hierarchical Clusterings. Journal of the American Statistical Association. 1983;78(383):553-69. doi: 10.1080/01621459.1983.10478008.
