# Supplementary material for: Functional geometry of auditory cortical resting state networks derived from intracranial electrophysiology
Source: PLoS Biol. 2023 Aug 31;21(8):e3002239. doi: 10.1371/journal.pbio.3002239 (PMC10499207; doi:10.1371/journal.pbio.3002239)
Supplement: Attachment — Submitted filename: Functional geometry - PLoSBiol - Reviewer response FINAL.docx [file pbio.3002239.s017.docx]

***Response to Reviewers’ Comments***

**Functional geometry of auditory cortical resting state networks derived from intracranial electrophysiology**

Matthew I. Banks *et al.*

*The authors would like to thank the Reviewers for their thoughtful and constructive comments; the manuscript has been revised accordingly. The Reviewers’ comments are shown below in regular text, followed by the authors’ responses in italics.*

*Note to all reviewers: we increased the number of bootstrap iterations from 10,000 to 100,000, which changed all of the p-values slightly in the paper. This was done to ensure that the error estimates were stable. For statistical tests that were in the original manuscript, this is the only change, unless noted specifically in comments below.*

Reviewer #1: In this article, Banks and colleagues investigate the geometry of auditory resting state networks derived from iEEG. They used Diffusion Map Embedding to represent recording sites in a low-dimensional space. This approach permits the authors to characterize some specificities of the auditory networks at three scales. At the fine scale, they show a segregation of auditory cortical sites. At the mesoscale, they report the cluster of a limbic auditory stream. At the macroscale, they found a greater heterogeneity for the language dominant hemisphere.
The innovative approach followed in this study is interesting and brings pertinent insights on the current states of the art of human brain network organization at multiple levels. Please find below my comments to improve the impact of the paper and alleviate potential issues.

Majors:

There are two important aspects of the study that might be criticized by the community: the data used for the analyses correspond to resting state data, and while the main analyses rely on functional connectivity the link with brain function is somehow indirect.
Resting state data does not correspond to (pro)active processing performed by the brain. We know that neural networks are shaped by the type of computation, that is they can change depending on task demand (e.g., attention, memory etc.). The pertinence of resting state is still a matter of debate for some researchers, and it would be appropriate to tackle this possible limitation, at least in the discussion.

*We thank the reviewer for raising this concern. We appreciate the importance of this ongoing debate, and began the Introduction with a brief review of the rationale behind this approach (Lines 78-80). We agree that a deeper dive in the Discussion is warranted to address this important issue. To that end, we have added a section in the Discussion titled “Investigating cortical network organization using resting state data” (Lines 551-574) that expands on the strengths and limitations of analyses based on resting state data.*

The geometrical organization described in the study might be subjected to change if participants would have been asked to perform different active tasks. Therefore, when it comes to relate the present results to function some assertion could be tone down or clarified. For instance, the use of the word 'functional', 'functionally' or 'processing hierarchy' might lead to some misinterpretation as it related to the measure and not to brain function.

*We appreciate this concern and we are happy to clarify what we mean by the term ‘functional’. We agree that there is a tendency for this term to become overloaded with meaning. ‘Functional’ is used in the literature in contexts that extend well beyond brain responses during specific tasks. For example, ‘functional connectivity’ refers to statistical relationships between brain signals and is most typically estimated from task-free data. Similarly, the term ‘functional geometry’ was coined previously in a study examining data-driven methods for registering brain data across subjects (Langs et al 2010) and has become a standard term for referring to the representation of neurophysiological data in embedding space (e.g., Huang et al 2023). We use the word in a similar vein. We have clarified this by making several changes in the manuscript.*

1. *We have edited the last paragraph of the introduction to clarify the basis for the term ‘functional geometry’ (Lines 133-138).*
2. *We have changed both instances of the phrase “processing hierarchy” to “cortical hierarchy”.*

The authors tend to assess functionality by using additional dataset in a subset of participants, where mono-syllabic words were presented in semantic categorization and tone target detection tasks. Yet, this analysis and the corresponding results are limited: the authors do not provide enough details about the different data types, the processing was focused on high frequency activity and the topology of the network was not investigated during these tasks.

*We thank the reviewer for raising this important issue. In line with the reviewer’s suggestion above, we had added these data to the manuscript to better understand the relevance of our findings from analysis of resting state data to auditory processing per se. These data were obtained in the course of several previous investigations in which monosyllabic word stimuli were presented as part of a target recognition task. We have added further details of these experiments to Methods (Lines 1089-1107).*

*High gamma band is typically used for characterizing auditory responses in intracranial recordings, as it is dominated by local spiking activity. These responses are measuring brain activity, rather than connectivity. By contrast, especially feedforward information flow has been shown to be maximal in gamma band (e.g., Bastos et al 2015).*

*We agree that investigating network topology during the task would be an interesting line of inquiry for a future study. However, it would be challenging to do so with the recorded data. The task was not designed to allow for accurate estimates of connectivity. That is, it is not straightforward to estimate connectivity in sensory regions in the presence of an external stimulus, because the stimulus itself will drive correlated activity within the network. Thus, to do so accurately would sufficiently long stimulus-free periods during each trial, which does not apply to the recorded data.*

(2) The main analysis is conducted on orthogonalized power envelope correlation in the gamma-band.
Additional analyses are performed on other frequency bands using either the same metric, or wPLI (not fully described in the materials and methods section).

*We apologize for this omission. In revising the manuscript, we decided to focus exclusively on band power envelope correlations, expanding our presentation of data from other frequency bands. We have removed the wPLI data from the manuscript as we did not feel it added substantially to the overall paper.*

Supplementary Figure 8 summarizes the correlation between the different approaches. Yet not much attention is brought on the differences, although the authors underlie its importance (lines 297-298, lines 325-326 or lines 549-551). For instance, the results in the beta band suggest the existence of different geometrical configurations (i.e., in the beta band the correlation with other bands is the lowest). Knowing that the beta band can reflect top-down process, as compared to bottom-up process possibly carried by lower and higher frequency bands, one may wonder if the geometry of auditory network changes with the type of process/task.

*We agree that further elaboration on band-specific organizational properties is warranted. To that end, we expanded our presentation of band-specific features of network topology in Figure 5. Our results (and those of other studies) suggest that specific nodes in the network take on an outsized role in coordinating information transfer between different subnetworks, i.e., serve as network hubs, and these nodes do so only for communication within specific frequency bands. In the original draft, we compared the results for theta and gamma. Now, we have expanded on our presentation of these data to include all bands studied, and present those new results in new Figure 5c.*

*In addition, we were intrigued by the reviewer’s suggestion that it might be possible to distinguish feedforward versus feedforward network organization by considering different bands. In both the auditory and visual systems, gamma band connectivity is associated with feedforward signaling, and beta/alpha bands are associated with feedback signaling (see revised text in Introduction, lines 86-88, for references):*

*“For example, cortico-cortical feedforward versus feedback information exchange occurs via band-specific communication channels (gamma band and beta/alpha bands, respectively) in both the visual[11-15] and auditory[16-19] systems.”*

*Throughout the paper, we now focus on gamma band, as before, but also present beta band results as a complement.*

*We have performed analyses of the relationship between connectivity and auditory responsiveness for different frequency bands. Those additional results are summarized here.*

*First, we have measured the relationship between the clustering results presented in Figure 4 and auditory responsiveness measured as high gamma band power responses to word stimuli, by fitting a linear model with early gamma responses as outcome and cluster membership as a categorical predictor. We found that the relationship was the strongest for high gamma, gamma, and theta bands, across a range of cluster thresholds (for 5 clusters, R^2^ = 0.69 high gamma, 0.70 gamma, 0.36 beta, 0.43 alpha, 0.75 theta; for 9 clusters, R^2^ = 0.84 high gamma, 0.73 gamma, 0.55 beta, 0.65 alpha, 0.76 theta); late gamma responses followed a similar pattern. In the auditory system, alpha suppression following an auditory stimulus is postulated to reflect a top-down signaling-driven release from inhibition (Klimesch et al., 2007). Conversely, an increase in alpha power is associated with inhibition of cortical activation by task-irrelevant sounds (Ahveninen et al., 2017).*

*Next, we measured the regional distribution of alpha suppression from the same dataset used in Figure 4a. We observed a similar relationship between clustering and alpha suppression as we did for high gamma responses (for 5 clusters, R^2^ = 0.62 high gamma, 0.67 gamma, 0.35 beta, 0.39 alpha, 0.70 theta; for 9 clusters, R^2^ = 0.76 high gamma, 0.68 gamma, 0.53 beta, 0.58 alpha, 0.72 theta): clustering based on high gamma, gamma, and theta band connectivity matched the distribution of alpha suppression the best, while alpha band connectivity exhibited a comparatively weak relationship to alpha suppression.*

*Thus, the network organization derived from resting state iEEG data does not simply reflect responsivity in the context of auditory stimulation. Instead, it reflects an intrinsic organization that underlies the processing of sensory information as well as myriad other perceptual and cognitive processes that arise both consciously and subconsciously.*

Moreover, the different metrics (magnitude or phase) do not have the same neurophysiological meaning.

*It is true that the two functional connectivity metrics are distinct, and represent distinct manifestations of neuronal coupling. However, both measures reflect underlying synaptic coupling, and thus would be expected to overlap. Indeed, the measures are reported to be correlated with each other both in our data and in previous studies (Mostame & Sadaghiani 2020) and thus the modest correlations that we observe are not surprising. All that being said, as we mention above we have decided to focus exclusively on band power envelope correlations, expanding our presentation of data from other frequency bands. We have removed the wPLI data from the manuscript.*

It would be critical for the community to approach these questions. Even if it is not the main goal of the study, measuring and reporting the malleability/rigidity of the geographical distances would certainly be beneficial for the impact of the paper.

*We agree that this is an important issue and we have expanded our presentation of results in different frequency bands and our discussion of those results. (See comments above, Figure 5c, Supplementary Figure 6, and Discussion section titled “Frequency band-specific properties of cortical networks”, Lines 576-597.)*

(3) The authors use fMRI data to, somehow, validate their results. It brings the question of the benefit of using iEEG rather than fMRI. How to convince the community about the advantage of using iEEG (i.e., clinical data difficult to get) rather than fMRI if the latest is used as a form of control and easier to get? The most immediate response would be the difference in time resolution. But this advantage is not obvious in the current state of the manuscript.

*We appreciate the reviewer raising this issue, as one of the novel aspects of this work is to apply DME analysis to electrophysiological data. Because of the superior temporal resolution of electrophysiology compared to fMRI, an advantage of investigations of resting state activity using electrophysiological data is that it is possible to apply the analysis to different frequency bands, which can distinguish networks associated with different functions. In response to the reviewer’s suggestions and as noted above, we have expanded our presentation of our results with different bands, and in particular point out findings in these bands that are distinct.*

*We have also addressed this issue by expanding our presentation of future directions in the Discussion (Lines 753-765). First, the superior time resolution of electrophysiological data allows for dynamic connectivity analysis on time scales relevant to sensory and cognitive processing. Second, an important next step for this work is to adapt this analysis to scalp EEG recordings, which offer considerable advantages over fMRI in terms of accessibility and cost. This type of data would have similar advantages over fMRI in terms of temporal resolution, for example allowing band-specific analyses.*

Also, it is known that the BOLD signal is positively correlated with iEEG gamma band activity and inversely correlated with alpha band activity. This might require more attention (e.g., in link with lines 406-408 or with Figure 7 panel d).

*We appreciate the reviewer’s careful attention to this detail. It is true that BOLD signal power is often reported to be inversely correlated with alpha band power (Ojemann et al 2013), but the same is not true for resting state functional connectivity (RSFC), which is the focus of the manuscript. That is, the correspondence between BOLD RSFC and iEEG band power RSFC indeed varies depending on frequency band (e.g., Hacker et al 2017; Kucyi et al 2018)., but inverse correlations are not typically observed. The variability in correlations across frequency bands presented here are consistent with these previous reports, and we have added these references to the suggested location. We note as well that there is regional heterogeneity to this correspondence in RSFC, and we are averaging the correlation values across these regions in Figure 7d. We have added text to the Discussion expanding on and clarifying this issue (Lines 576-597).*

(4) The authors wrote (line 324) that the results are consistent with network organization depending on temporal scale. It would be pertinent to further develop this argumentation as it is in direct link with the main characteristic of iEEG, its high temporal resolution. Maybe clarifying the meaning behind parameter "t" would be important (in the main text not in the supplements). Making links with the works on brain temporal time scales might be relevant too.

*We thank the reviewer for raising this issue. The parameter ‘t' does not relate to the temporal resolution of the analysis; rather, it sets the spatial scale of the analysis, i.e., the scale of the diffusion process on the graph that represents the brain network. It is akin to how close a viewer positions themselves to the plotted data cloud. At smaller values of ‘t’, the local details of relationships in the data cloud can be ascertained; larger values of ‘t’ corresponding to a more distant view, dimensions corresponding to small eigenvalues collapse and any variability along those dimensions is lost. In an earlier version of the manuscript, we included analyses of the data at different values of ‘t’, but the main result of those analyses was that the variability along dimension 1, e.g., the large distance between auditory cortex and prefrontal cortex, is a dominant feature of the data. Because we are able to make this observation solely with the analysis at a value of t = 1, while also discerning interesting organizational features of the data along other dimensions, we chose to present only the data for t = 1. We have clarified the meaning of the parameter ‘t’ in the Methods in the main manuscript document (Lines 981-989).*

(5) Somehow, this article 'straddles' between being a purely methodological paper and a study on brain network geometry. That is, it is not clear if this study is a proof of concept of a method (i.e., Diffusion Map Embedding) applied to iEEG or an unprecedented investigation of the organization of auditory brain network. These two aspects of the paper are not mutually exclusive, but maybe this could be further delineated throughout the manuscript.

*We appreciate this suggestion. We have added language throughout to highlight the novel aspects of both our approach and our results. Examples include the following. Introduction: Lines 102-103, 138-148; Results: Lines 231-235, 380-381.*

Minors:

- In the introduction is mentioned the fact that investigation of the anterior temporal lobe with fMRI is subjected to methodological issues, but the ANT is not the focus of the present study.

*We agree it is important to clarify this point. The anterior temporal lobe contains important nodes in speech and language networks, as illustrated in Figures 4 and 5. Thus, while the anterior temporal lobe is not the sole focus of the paper, ROIs within this region (e.g., temporal pole, anterior portions of superior, middle, and inferior temporal gyri) do figure prominently in the results presented here, findings made possible in part because of iEEG signal quality in those regions. The same is true for the upper versus lower banks of the superior temporal sulcus. We have modified the text in the Introduction to clarify this point. (Lines 88-92).*

-  In the introduction, when different auditory areas are described the concepts of specificity and sensitivity should be clarified.

*Thank you for this suggestion. We agree that the concepts of response specificity and sensitivity are relevant to functional parcellation of auditory cortex. For example, HGPM activity is sensitive to all kinds of sounds whereas PT is more specific to speech (e.g., Hamilton et al., 2021). We have added a statement relating these concepts to underlying inter-regional connectivity (e.g., Abrams et al., 2020) and provided appropriate references (Lines 104-107).*

- The introduction finishes with a long sentence that is not easy to understand.  Maybe it could be related to the results of the study.

*Thank you for bringing up this issue. The sentence has been revised.*

- Information is lacking concerning the methods used to normalize data into the template(s) (e.g., MNI, atlases…).

*We have included additional detail regarding our anatomical reconstruction approach (lines 838-848).*

- In Figure 1, the purplish colors for the Auditory core and sensori-motor regions are not easy to distinguish.

*Thank you for the comment. We have changed the color used to denote Auditory core cortex to a shade of orange, to better align it with other canonical auditory cortical ROIs and to avoid the potential confusion with sensorimotor ROIs.*

- The variance between participants should be reported (e.g., in the functional geometry of cortical networks).

*We agree that this variance is important to report. For Figure 3B, Supplementary Figure 3, and Supplementary Movies 1 & 2, the variance across participants obtained via bootstrap is shown by the size of the ellipsoid for each ROI. Resampling was used because we cannot compare embeddings in different participants directly due to variable electrode coverage. We have clarified this in the text (lines 219-221, 1042-1056) and in the caption for Figure 3.*

- Can the authors add the comparisons between dimensions 2 & 3, 3 & 4 and 2 & 4?

*We currently show these comparisons in Supplementary Figure 3. We are happy to move this figure to the main manuscript if the reviewer wishes, though we would need an exception from the editor to the limit on display items.*

- line 202, maybe the term "phase" could be replaced by 'time-locked' or nothing that would permit to avoid confusion with the phase of the signal which is not specifically analyzed here.

*The term “phase” does in fact refer to the phase of the signal. Connectivity at zero phase lag represents instantaneous connectivity, which will occur only if the correlations are due to volume conduction (rather than through brain circuits) and is thus spurious, or if the two regions are driven at identical latencies by a third region, but are not themselves connected. In either case, it is prudent to remove those correlations from the data before analyzing connectivity.*

- What is the difference between the terms 'polysensory' (line 226), multimodal (line 248) or 'transmodal' (line 541, 548)? if they convey the same meaning, the same term must be used.

*We apologize for the ambiguity in our use of these terms. The terms “polysensory” and “multimodal” are indeed similar in meaning, and distinguishing between these terms does not add to the paper. We have chosen to keep the single instance of the term ‘polysensory’, as it refers to sensory processing in InsP that is not restricted to one sensory modality. We have replaced the two instances of the term ‘multimodal integration’. In the first, it has been replaced with “integration of sensory information from multiple modalities”. In the second, it has been replaced with the term ‘transmodal’, which refers to regions in which activity changes are not tied to specific sensory modalities (Mesulam 1998; Braga & Leech 2015).*

- The PP and InsP are not easily visible in Figure 3. Because those regions are discussed in detail, they could be somehow highlighted in the figure.

*We have modified Fig. 3 to eliminate the overlap between PP and InsP labels and make them more easily visible.*

- line 251, it is said that the brain parcellation in Figure 1 is suboptimal because there are no quantitative criteria for designating 'auditory-related' and 'auditory non-core'. But these areas are color coded in green and yellow, respectively. Maybe this can be clarified?

*We apologize that this was not clear in the previous version. We have changed the text of that paragraph to the following (lines 301-309):*

*“This heterogeneity, as well as the embedding locations of PP and STSU, suggests that DME can be used to improve the brain parcellation scheme from Figure 1. For instance, MTGA in that scheme was labeled as part of the ‘Auditory-related’ group based on its location on the lateral temporal convexity and its anatomical proximity to canonical auditory cortex. The ‘Other’ group contains a large and diverse collection of ROIs whose relationship to auditory structures and speech and language processing is unclear. A more principled approach is warranted to arrange these and other ROIs into functional groups or streams based upon their physiology. One approach to developing such a data-driven parcellation scheme is to apply hierarchical clustering to the data in embedding space.”*

- In Figure 4 panel b, to facilitate the comparison dashed lines extending the x-axis along the y-axis would be useful. In panel c, the colors coding is not enough contrasted to distinguish the different clusters.

*Thank you for these suggestions. We have added vertical grid lines to Figure 4a. We also re-colored the clusters in Figure 4 to make them easier to distinguish from one another.*

- In supplementary Figure 5, it would be nice to also have the derived brain parcellation based on hierarchical clustering (like in Figure 4).

*As part of our expansion of the presentation of results from other frequency bands, we have replaced Supplementary Figures 4 and 5 with a new Supplementary Figure 6 showing dendrograms from all studied bands. We feel that adding new display elements showing brain parcellations for all bands would greatly expand the number of display elements without substantially enhancing the presentation of these data.*

- lines 302-304: Actually, there are tools/metrics, from graph theory that define centrality and strong connectivity. The corresponding sentence should be revised accordingly.

*Thank you for this point; the sentence has been revised accordingly.*

- line 312-313 and Figure 5b, it is not clear how the distance from the center could not be inversely proportional to the connectivity metric used. How would the authors explain a scenario where it would not be the case (as one measure is derived from the other)?

*We have clarified this idea with new text (lines 389-397) in the presentation of Figure 5, which has also been expanded to show the results from all frequency bands.*

- In Figure 5 panel b, it is not clear how the four dimensions are collapsed.

*The distance calculated is Euclidean distance. This has been clarified in the text (lines 392-394) and in the figure caption.*

- In Figure 7 panel C, there is a cloud of points showing a horizontal trend, can the authors provide information about it?

*All of the points in that cloud with a horizontal trend correspond to pairwise distances with dPreCG. Here, the location of dPreCG in the iEEG embedding space is somewhat remote from the rest of the data cloud, likely reflecting connectivity that is distinct from other sampled ROIs. This is apparent in the average data of Figure 3 as well. This results in low variance of edge distances involving this ROI. This is less apparent in the fMRI data (see Figure 7a); hence, the edge distances vary as for other ROIs. We do not believe this reflects poor sampling of this ROIs edges (connections) with other ROIs – in that sense dPreCG is about average.*

- lines 460-461, what are the data-driven hypotheses for future studies?

*These hypotheses refer to results of exploratory analyses presented in the paper. We have clarified this in the text (lines 547-549).*

- Some considerations about the visual and sensory-motor areas would nicely complement the discussion on FG and MOG on multisensory integration.

*We are grateful to the reviewer for this suggestion. We have added text placing the proximity of sensorimotor ROIs to the dorsal cluster and the proximity of visual ROIs to the ventral cluster within the context of visual and sensorimotor contributions to ventral and dorsal stream processing, respectively (lines 669-673).*

- line 662, the length of the data is really 13 +/- 11 min?

*We have replaced this text with a more meaningful summary of the length of recordings: “In all but two participants, recording durations were between 10 and 18 minutes, the median was 10; in one participant duration was 6 min., and in one participant the duration was 81 min.”*

- How does electrodes in the white matter were identify?

*This has been clarified in Methods (lines 876-879): “Depth electrode contacts localized to the white matter were also excluded. Location within cortical white matter was determined based on visual inspection of anatomical reconstruction data (MRI sections along sagittal, coronal, and axial planes) as done in our previous studies (e.g., [62]).”*

- lines 740-744, the procedure used to avoid volume conduction is described without explaining how the use of high frequency activity can help to get rid of passive signal spread that dominate low frequencies.

*We apologize that this was not clear. We have modified the relevant text in Methods as follows (lines 916-921): “On occasion, noise from in-room clinical equipment and muscle artifacts appeared in the data as shared signals across channels. These types of noise are typically broadband, and can be detected via analysis of frequencies higher than those of interest here. To remove these signals, data from retained channels were high-pass filtered above 200 Hz, and a spatial filter was derived from the singular value decomposition omitting the first singular vector. This spatial filter was then applied to the broadband signal to remove this common signal.”*

- line 776, the rationale for using the top-third connections is missing.

*We have added rationale for the thresholding method employed (lines 955-962).*

- In supplementary table 1, please add the number of ECoG electrodes and sEEG electrodes per participants.

*The number of subdural sites (i.e., ECoG) has been added to Supplementary Table 1. The rest of the sites in each participant is the number of sEEG sites.*

- In the legend of supplementary Figure 2, it seems that 'noise' should be SNR.

*“superior noise” has been changed to “superior SNR”*

Reviewer #2: This is a very interesting report on a large data set of invasive (stereotactical EEG) recordings in human patients, using resting-state data to establish/understand better potential topological relations in a dimensionality-reduced network representation of these data (all being based on gamma-band power envelopes; see specific comments on this choice below).
The paper shares with many other topological analysis papers its pro's and con's: It goes far beyond other iEEG/intracranial studies in that it has larger coverage and larger N than many other studies before and thus is able to describe the data at a more abstract level. (but see statistical concerns on pooling data from participants below.) As a problem arising from this, it remains unclear (to me at least) what the achived level of description really adds beyond known anatomical hierarchical relations, in lieu of functional data and/or behavioural markers. The paper as such feels somewhat premature and the data might better serve as an atlas-like basis for future functional interrogations.
In sum, I was intruiged but ultimately not convinced about a new mechanistic insight being conveyed.

More detailed comments follow below, page numbers referring to the PDF made available by Plos Biol:

[page 22]: I overall struggled to see the deeper utility of the present data. I am not questioning the merit of iEEG with cortex-wide coverage for our progess in understanding cortical functional organisation. However, the present data use resting-state data to make in places somewhat ciurcular conclusions from the anatomical structures where electrodes were placed to the functions ascribed to the regions.
Clear new insights are sparse from these analyses, accordingly. Their value might lie more in a serving as a descriptive, atlas-like basis for future studies and the data should be made publicly available, accordingly.

*We agree that this is not a purely data-driven analysis, and we are relying in part on results of previous studies. Our goal was to provide framework that can serve to refine our understanding of the functional identities of the studied regions. Indeed, with this approach we were able to make several discoveries that were not expected or implied, including, for example, relationships of the upper and the lower bank of the superior temporal sulcus to canonical auditory cortex and areas involved in semantic processing. We were also able to identify network hubs and test hypotheses regarding laterality of speech and language regions. We will make the data publicly available to serve as a basis for future analysis.*

[page 17]: Pooling across participants: To the best of my understanding, the analyses as reported do not take particular care of the hierarchical structure of these data. The network analyses seem to rest on pooled data across electrodes and participants.
This essentially equals what could be referred to as a "fixed effects" analysis.
It is unclear how a generalisation across electrodes/participants sampled is actually feasible here.

*We apologize that this was not clear in the original manuscript. The bootstrapping for Figure 3 (and throughout the paper) did preserve the hierarchical structure of the data, i.e., resampling subjects with replacement. The relevant section in Methods has been revised (lines 1040-1051). That section is as follows: “Estimating precision in position and distances in embedding space. To obtain error estimates for both ROI locations in embedding space and embedding distance between ROIs, average ROI × ROI adjacency matrices were calculated. Our data are hierarchical/multilevel, in that we sampled participants in whom there are multiple recording sites. Nonparametric bootstrap sampling at the highest level (“cluster bootstrap”[159]; here the word “cluster” refers to the hierarchical/multilevel structure of the data, with multiple recording sites within participants, rather than algorithmic clustering) is the preferred approach for hierarchical data when groups (here, participants) are sampled and observations (here, recording sites) occur within those groups[160], with fewer necessary assumptions than multilevel (mixed-effects) modelling (e.g., subject effects are not assumed to be linear). Using this approach, participants were resampled with replacement, connectivity averaged across the bootstrapped samples, and diffusion map embedding performed for 100,000 such adjacency matrices. For locations in embedding space, these embeddings were then mapped via the change of basis procedure described above to the original group average embedding space. For each ROI, the mapped bootstrap iterations produced a cloud of locations in embedding space that were summarized by the standard deviation in each dimension. For embedding distances, no change of basis was necessary because distances are preserved across bases.”*

[page 23]: The rationale of working off gamma-band envelopes when building/analysing large-scale brain-wide networks remained somewhat elusive. The authors argue more in passing than anything (line 294f and supplements) that there was an overlap with networks derived from the (in my mind more plausible for long-range connections) theta-band envelopes, but the differences and similarities are not further explained.

*We apologize that this was not clear. We have clarified our motivation for focusing on gamma- (and now additionally beta-) band connectivity (lines 86-88, 169-172; 583-587). We have also expanded our presentation of results from other frequency bands (Figure 5; Supplementary Figure 6).*

Neither do the authors discuss how gamma-band envelopes could become a signifier of long-range connectivity. There must be some form of subcortical or otherwise mediated anatomical pathways. This would probably need to be developed and discussed further in the discussion section.

*We appreciate the reviewer’s attention to this important issue. It is true that for measures that rely on phase synchronization, gamma frequency connectivity would reflect primarily local connections. However, the measure that we use to quantify connectivity for most of the results presented here is based on power envelope correlations over slow time scales. Previous reports have shown that long-range connections manifest in this form of functional connectivity (e.g., Hipp et al 2012; Hacker et al., 2017).*

[page 25]: The analysis of language-dominant vs non-dominant hemisphere is interesting and laudable. Statistically speaking, however, the analysis would deserve proper control for potential other, confounding differences between the two groups of patients.

*We agree that the patients’ demographic and clinical background (including Age, Sex, Coverage, Seizure focus, as detailed Table S1) might constitute potential confounding factors. We examined this and did not find any systematic differences between participants with implants in dominant vs. non-dominant hemispheres. For example, comparisons between the two groups of participants (in which recordings were obtained from the language-dominant and non-dominant hemisphere, respectively) did not reveal significant differences in terms of age (p=0.454, Wilcoxon rank sum test), sex (p=1.000, Fisher exact test), or type of implantation surgery (i.e., stereo-EEGor craniotomy for placement of subdural arrays or) (p = 1.000, Fisher exact test).*

[page 27]: By and large, the paper appears to argue that the frequency band under consideration is not crucial. This again would also be a somewhat surprising result and would deserve much better  documentation/justification than the few sentences about alpha, beta and theta currently provide.

*We did not intend to downplay the importance of frequency band for information exchange in the brain. We note here and in the paper, however, that previous reports have found considerable overlap in network organization across frequency band (e.g., Kucyi et al 2018), likely because signals tend to be correlated across frequency. Our intention was to demonstrate that the method can be readily applied in all bands tested, and that the results are consistent. We agree that this important issue merits a more thorough treatment, and have expanded our presentation of results from other frequency bands (Figure 5c, Supplementary Figure 6).*

There is a large body of work, also in this journal (e.g. in recent years Keitel et al., Kluger et al, Alavash et al, in other journals eg Hipp et al, Nat Neurosci) documenting frequency-specific "fingerprints" of different cortical areas. Also in functional networks derived from fMRI data, (much slower) frequency-specific patterns have been exposed. How do the authors reconcile their non-specificity for their DME networks with these findings? (See also my concern about gamma-band envelopes for estimating long-range connections more generally.)

*Thank you for this comment. As we mention above, in our effort to make the case that the method presented here is particularly useful for electrophysiological data because it can be applied in to all frequency bands, we did not emphasize enough the importance of band-specific results. We have expanded the presentation of these results in response to queries from both this reviewer and reviewer #1 (Figure 5c, Supplementary Figure 6).*

[page 32]: The analyses targeting specific hierarchical relations, e.g. within STS, exposed another problem with the general approach of the present manuscript: The authors do not demonstrate that the found network solutions (ie., clusterings in embedding space) are robust across cross-validated subsets of the data. To the best of my reading, there are no such robustness analyses being reported?

*We are grateful to the reviewer for requesting clarification of this important point. We are not using the clustering results for hypothesis testing; rather, they are hypothesis- generating. However, we agree that our frequent use of the term ‘cluster’ outside the context of the clustering analysis was confusing. We have revised the text accordingly. When we compare STSU and STSL to auditory cortical ROIs and to semantic processing ROIs, we are basing those comparison groups on previous reports from the literature. We then use those prior results to test hypotheses about STSL and STSU in embedding space, not relative to the clustering analysis of Figure 4. Robustness in that analysis is assessed using permutation testing, which is an effective and flexible method (i.e., non-parametric and relatively free of assumptions) for hypothesis testing. The permutation testing method respects the hierarchical structure of the data, as we are permuting the labels at the subject level.*

*We also used bootstrapping to evaluate the robustness of the clustering results presented in Figure 4 and Supplementary Figure 6 via two approaches. Overall stability of the clustering scheme was evaluated using a method for comparing the similarity of two clustering results, the Fowlkes-Mallow index (Fowlkes and Mallows 1983). The results of the analysis as a function of threshold and frequency band are shown in Supplementary Figure 7.*

*The consistency across cluster number reinforces the idea that, as in any cluster analysis, it is difficult to select a single cluster number based solely on the data being clustered. For example, this is a common finding in brain parcellation schemes based on functional connectivity (e.g., Yeo 2011; Schaefer 2018). The data are spatially and temporally scale free over a wide range of these parameters. We have revised the presentation of the results in Figure 4 and new Supplementary Figure 6 to emphasize this important point. We now show the results of two cluster thresholds, and show how the results relate to each other across these thresholds.*

*For gamma band data, we also estimated cluster-wise stability using the Jaccard Coefficient. These results are presented as a table in Supplementary Figure 7.*

MINOR:
[page 12] The introduction should do more to add justification and specificity: Why the diffusion map embedding (vs many other dimensionality-reduction and graphing algorithms)?
For a paper striving to provide a definitive answer, the methodological choices (for which there were plenty here) are obviously inseparable from the conclusions to be drawn. As a consequence, major choices like the algorithm become a key feature not a side note and should be justified and interpreted accordingly.

*We thank the reviewer for raising this issue. DME is a part of a broader class of methods that rely on spectral analysis of similarity matrices to reveal underlying structure in a dataset. It is also being used more and more frequently in the analysis of resting state imaging data. The is the first time it is being applied to electrophysiological data. We have added text to the Introduction to clarify this (lines 131-135, 145-146).*

[page 13]: The sentence in line 133f. needs a citation. ("… auditory processing").

*We have added references to our previous studies (Nourski et al., 2018a,b; Banks et al., 2020; Nourski et al. 2021a,b,c) that utilized this anatomical parcellation scheme (lines 162-164).*

Reviewer #3: Review of PBIOLOGY-D-22-02638R1, "Functional geometry of auditory cortical resting state networks derived from intracranial electrophysiology"
In this article, the authors perform a series of analyses to determine the organization of auditory and auditory-adjacent areas in the brain based on their patterns of resting state functional connectivity obtained via intracranial EEG. The overall dataset represents a substantial number of subjects, recording sites, and time points when amassed together. The analyses are reported in a coherent theoretical and empirical framework. Most importantly, the results provide important new insight into the functional organization of the auditory system and, in particular, its integration with the rest of the brain. The is particularly interesting and important when considering the role of audition in the human faculty of language. I note from the manuscript number that this paper is a revision, but want to acknowledge that I am a new reviewer seeing this manuscript for the first time.

This report has numerous strengths. The sample size and density is one. The clear prose and beautiful visualizations is another. In addition, I thought the hemispheric asymmetry analyses were particularly interesting and well executed. Finally, a great strength of the paper is the direct comparison between the iEEG data and the rs-fMRI data from the same participants. Overall, the paper is an empirical and methodological tour de force. Broadly, I think it will make an important contribution to the literature in this area, and will inspire future work based on these methods in other domains.

I have a number of only small comments / recommendations regarding the framing of certain sections:
1) In the Introduction and throughout the paper, I think the claims about lateralization of speech and language could be made with some more nuance. While neurological and neuroimaging evidence do point consistently and uniformly to the leftward lateralization (necessary and sufficient) for language, most evidence, both neurological and neuroimaging, seems to suggest a bilateral organization of speech (perception / recognition). This is most clearly explained in the Hickok & Poeppel (2007) model. Some more nuance in the introduction of this idea could help the paper not come across as overly dogmatic or naive about the bilateral organization of speech.

*We thank the reviewer for this suggestion. We have added nuance to the text in the Introduction and included the Hickok reference as suggested (lines 124-129).*

2) In the Discussion, I think an important caveat to acknowledge is that the patterns of organization are based on resting state data. It remains an open and unexplored question whether and how these networks are also attested during tasks. There is a lot of thought recently in the literature on dynamic network brain (re)organization / reconfiguration during different kinds of tasks and their difficulty. This is not a criticism of the approach, but it is important to note that these networks are based on a snapshot of behavior (rest) rather that the full, complex range of auditory behaviors.

*We agree that this is an important point to emphasize. We have added text to the discussion about resting state network organization and its dynamic reconfiguration during task-related activity (lines 570-574).*

3) Another minor question in the discussion centers on the hub/spoke distinction. The authors do a good job framing their findings regarding the Hubs, but the brain areas identified as Spokes are not discussed. I wonder if they might comment also on why certain regions (principally somatomotor regions, but also primary auditory regions) appear to be "spokes" in their network organization.

*We thank the reviewer for pointing out that this was unclear. Hubs are nodes that receive sensory information from spokes and send information to executive and motor areas. We have changed the text in Results that introduces this analysis to make this clear (lines 377-378).*

4) Finally, with regard to the discussion, the authors note differences in the sparseness of network organization in left vs. right hemispheres, and that these are not exclusive to language ROIs. I wonder if they might discuss more about non-linguistic (i.e., domain general) differences in hemispheric functional organization. For instance, classically, a great deal has been made about local vs. global processing in the left vs. right hemisphere, both in vision, space, navigation, etc.

*We are grateful to the reviewer for this excellent suggestion. We have added text to the Discussion focused on the possibility that asymmetry of domain-general neural systems may contribute to the broader hemispheric asymmetry that we observed (lines 726-731).*

All of these points are quite minor and none of them stand in the way of publishing this impressive work. I learned a great deal reading this paper and think it makes a tremendous contribution to the literature. I want to congratulate the authors on their hard work, compelling findings, and beautiful presentation of these data.

***Cited references***

*Abrams DA, Kochalka J, Bhide S, Ryali S, Menon V. Intrinsic functional architecture of the human speech processing network. Cortex. 2020;129:41-56. Epub 2020/05/20. doi: 10.1016/j.cortex.2020.03.013. PubMed PMID: 32428761.*

*Ahveninen J, Seidman LJ, Chang WT, Hämäläinen M, Huang S. Suppression of irrelevant sounds during auditory working memory. Neuroimage. 2017;161:1-8. doi:10.1016/j.neuroimage.2017.08.040*

*Banks MI, Krause BM, Endemann CM, Campbell DI, Kovach CK, Dyken ME, et al. Cortical functional connectivity indexes arousal state during sleep and anesthesia. Neuroimage. 2020;211:116627. Epub 2020/02/12. doi: 10.1016/j.neuroimage.2020.116627. PubMed PMID: 32045640; PubMed Central PMCID: PMCPMC7117963.*

*Bastos AM, Vezoli J, Bosman CA, Schoffelen JM, Oostenveld R, Dowdall JR, et al. Visual areas exert feedforward and feedback influences through distinct frequency channels. Neuron. 2015;85(2):390-401. doi: 10.1016/j.neuron.2014.12.018. PubMed PMID: 25556836.*

*Braga RM, Leech R. Echoes of the Brain: Local-Scale Representation of Whole-Brain Functional Networks within Transmodal Cortex. Neuroscientist. 2015;21(5):540-551. doi:10.1177/1073858415585730.*

*Fowlkes, E. B., & Mallows, C. L. (1983). A method for comparing two hierarchical clusterings. Journal of the American statistical association, 78(383), 553-569.*

*Hacker CD, Snyder AZ, Pahwa M, Corbetta M, Leuthardt EC. Frequency-specific electrophysiologic correlates of resting state fMRI networks. Neuroimage. 2017;149:446-57. Epub 2017/02/06. doi: 10.1016/j.neuroimage.2017.01.054. PubMed PMID: 28159686; PubMed Central PMCID: PMCPMC5745814.*

*Hamilton LS, Oganian Y, Hall J, Chang EF. Parallel and distributed encoding of speech across human auditory cortex. Cell. 2021;184(18):4626-39 e13. Epub 2021/08/20. doi: 10.1016/j.cell.2021.07.019. PubMed PMID: 34411517; PubMed Central PMCID: PMCPMC8456481.*

*Hipp JF, Hawellek DJ, Corbetta M, Siegel M, Engel AK. Large-scale cortical correlation structure of spontaneous oscillatory activity. Nat Neurosci. 2012;15(6):884-90. Epub 2012/05/09. doi: 10.1038/nn.3101. PubMed PMID: 22561454; PubMed Central PMCID: PMCPMC3861400.*

*Huang Z, Mashour GA, Hudetz AG. Functional geometry of the cortex encodes dimensions of consciousness. Nat Commun. 2023;14(1):72. Epub 2023/01/06. doi: 10.1038/s41467-022-35764-7. PubMed PMID: 36604428; PubMed Central PMCID: PMCPMC9814511.*

*Klimesch W, Sauseng P, Hanslmayr S. EEG alpha oscillations: the inhibition-timing hypothesis. Brain Res Rev. 2007;53(1):63-88. doi:10.1016/j.brainresrev.2006.06.003*

*Kucyi A, Schrouff J, Bickel S, Foster BL, Shine JM, Parvizi J. Intracranial Electrophysiology Reveals Reproducible Intrinsic Functional Connectivity within Human Brain Networks. J Neurosci. 2018;38(17):4230-42. Epub 2018/04/08. doi: 10.1523/jneurosci.0217-18.2018. PubMed PMID: 29626167; PubMed Central PMCID: PMCPMC5963853.*

*Langs G, Golland P, Tie Y, Rigolo L, Golby AJ. Functional Geometry Alignment and Localization of Brain Areas. Adv Neural Inf Process Syst. 2010;1:1225-33. Epub 2010/01/01. PubMed PMID: 24808719; PubMed Central PMCID: PMCPMC4010233.*

*Mesulam MM. From sensation to cognition. Brain. 1998;121 ( Pt 6):1013-1052. doi:10.1093/brain/121.6.1013;*

*Mostame P, Sadaghiani S. Phase- and amplitude-coupling are tied by an intrinsic spatial organization but show divergent stimulus-related changes. NeuroImage 2020. 219:117051,* [*https://doi.org/10.1016/j.neuroimage.2020.117051*](https://doi.org/10.1016/j.neuroimage.2020.117051)

*Nourski KV, Steinschneider M, Rhone AE, Kawasaki H, Howard MA, 3rd, Banks MI. Auditory Predictive Coding across Awareness States under Anesthesia: An Intracranial Electrophysiology Study. J Neurosci. 2018;38(39):8441-52. Epub 2018/08/22. doi: 10.1523/JNEUROSCI.0967-18.2018. PubMed PMID: 30126970; PubMed Central PMCID: PMCPMC6158689.*

*Nourski KV, Steinschneider M, Rhone AE, Kawasaki H, Howard MA, 3rd, Banks MI. Processing of auditory novelty across the cortical hierarchy: An intracranial electrophysiology study. Neuroimage. 2018;183:412-24. Epub 2018/08/17. doi: 10.1016/j.neuroimage.2018.08.027. PubMed PMID: 30114466; PubMed Central PMCID: PMCPMC6207077*

*Nourski KV, Steinschneider M, Rhone AE, Krause BM, Kawasaki H, Banks MI. Cortical responses to auditory novelty across task conditions: An intracranial electrophysiology study. Hear Res. 2021;399:107911. Epub 2020/02/23. doi: 10.1016/j.heares.2020.107911. PubMed PMID: 32081413; PubMed Central PMCID: PMCPMC7417283.*

*Nourski KV, Steinschneider M, Rhone AE, Krause BM, Mueller RN, Kawasaki H, et al. Cortical Responses to Vowel Sequences in Awake and Anesthetized States: A Human Intracranial Electrophysiology Study. Cereb Cortex. 2021;31(12):5435-48. Epub 2021/06/13. doi: 10.1093/cercor/bhab168. PubMed PMID: 34117741; PubMed Central PMCID: PMCPMC8568007.*

*Nourski KV, Steinschneider M, Rhone AE, Mueller RN, Kawasaki H, Banks MI. Arousal State-Dependence of Interactions Between Short- and Long-Term Auditory Novelty Responses in Human Subjects. Front Hum Neurosci. 2021;15:737230. Epub 2021/10/19. doi: 10.3389/fnhum.2021.737230. PubMed PMID: 34658820; PubMed Central PMCID: PMCPMC8517406.*

*Ojemann GA, Ojemann J, Ramsey NF. Relation between functional magnetic resonance imaging (fMRI) and single neuron, local field potential (LFP) and electrocorticography (ECoG) activity in human cortex. Front Hum Neurosci. 2013;7:34. Epub 2013/02/23. doi: 10.3389/fnhum.2013.00034. PubMed PMID: 23431088; PubMed Central PMCID: PMCPMC3576621.*
