# Supplementary material for: Functional geometry of auditory cortical resting state networks derived from intracranial electrophysiology
Source: PLoS Biol. 2023 Aug 31;21(8):e3002239. doi: 10.1371/journal.pbio.3002239 (PMC10499207; doi:10.1371/journal.pbio.3002239)
Supplement: Attachment — Submitted filename: Functional geometry - PLoSBiol - Rev2 - Rev resp FINAL.docx [file pbio.3002239.s018.docx]

**Authors’ Response to Reviewers’ comments**

**Manuscript title:**

Functional geometry of auditory cortical resting state networks derived from intracranial electrophysiology

Matthew I. Banks_,_ Bryan M. Krause, D. Graham Berger, Declan I. Campbell, Aaron D. Boes, Joel E. Bruss, Christopher K. Kovach, Hiroto Kawasaki, Mitchell Steinschneider, Kirill V. Nourski

The authors would like to thank the Editor and the Reviewers for their thoughtful and constructive comments. We have thoroughly revised the manuscript to address Reviewer #1’s remaining critiques and suggestions. The Reviewer’s comments are shown below, followed by the authors’ responses in italics.

**Reviewer #1:**

The authors made substantial changes to the manuscript. While they strengthen the quality of the study, there is still some concerns that must be taken into account. Please find here some suggestions.

(1) Pertinence of resting state data.
Based on the adding made since the last version of the manuscript, some readers may think that event related paradigms have only cons when it comes to estimate functional connectivity. Although, that are many valuable publications that investigated functional connectivity with active protocols. The sections focusing on the pros of resting states data should be revisited accordingly. To defend their point of view, the authors could explain in the manuscript why they did not analyzed the event-related data to compare them with the resting state result.

*We thank the Reviewer for raising this important point. We have added text to the Discussion (L600-602) to clarify that connectivity can also be estimated from task-based activity, and that these analyses have provided important insights into brain function:*

*“Connectivity can also be measured in the context of event-related paradigms, including behavioral tasks [3, 100]. These analyses have provided important insights into the relationship between network structure and function [101], especially in the context of attention [102].”*

(2) Functional connectivity metric.
The use of band-pass filter after estimating the power of each frequency band (line 1062) focuses the signal on the slow fluctuation of each band. This smoothing may have some impact on the present results (e.g., link with cross frequency coupling) or may explain why the results from different frequency bands present some similarities.
As the authors performed the same series of analysis with wPLI, it is worth reporting them, at least in the supplements.

*We have added the wPLI results back to Supplementary Figure 5, as the reviewer has requested. Text has been added to the Results section describing these results (L255-260):*

*“A second connectivity measure based on phase synchronization (debiased weighted phase lag index, wPLI [70]) produced embeddings in the alpha and theta bands that were similar to each other (r = 0.85; Supplementary Fig. 5b) and to those derived from envelope correlations in those bands (r ≥ 0.73), suggesting that common features of network structure are captured by DME. Interestingly, correlations were much lower for beta-band wPLI, indicating that a phase-based connectivity measure captures distinct features in this band.”*

(4) Threshold.
Some thresholds applied to connectivity matrices are subjective (lines 1066-1073 and lines 1092-1102). It would be critical to provide an objective estimate of the influence of these thresholds.

*We agree with the Reviewer that evaluating sensitivity of the results to these parameter choices is important. The two parameters the reviewer references are the threshold for connectivity matrices (chosen to be 33%) and the value of the diffusion parameter t (chosen to be t = 1). We have explored sensitivity to these parameter choices in two ways.*

*First, we calculated correlations between inter-ROI distances obtained from embeddings with five different thresholds ranging from 10 – 50%, and with the embedding obtained with t = 1 compared to the embedding obtained from the multiscale implementation of DME, in which all values of t are considered simultaneously. This sensitivity analysis is similar to the analysis already in the manuscript evaluating choice of frequency band and measure (Supplementary Figure 5b). We present the correlations across threshold in Supplementary Figure 5a, and the correlation between t = 1 and multiscale in the text. The latter correlation is r = 0.94, indicating low sensitivity to the specific choice of t. For the threshold parameter, correlation values for adjacent threshold values were r* ≥ *0.87, and even comparing the two extreme threshold values (10% versus 50%), the correlation is still substantial (r = 0.64). We added text to the Results (L240-249) describing these analyses:*

*“The data shown in Figure 4 are based on a value of the diffusion parameter t = 1, chosen to maximize the local network structure that can be detected in the data. To test the sensitivity of the results to this parameter choice, the analysis was repeated using the ‘multi-scale’ version of DME, which considers all values of t simultaneously [69] (see Methods and Appendix 1). The results were compared to those in Figure 4 by calculation the correlation of inter-ROI distances in the two analyses. The correlation was extremely high (r = 0.94), indicating that the results are robust to the specific choice of the parameter t. Correlation values were also high for results obtained with connectivity thresholds ranging from 10-50% (Supplementary Fig. 5a), indicating that the results were also robust to the specific choice of threshold.”*

*Second, we present the hierarchical clustering results for these five threshold values and for the mulstiscale DME with threshold = 33% in Supplementary Figure 7. These results show that the same ROIs that cluster together in embeddings obtained with t = 1 and threshold = 33% also cluster together for other threshold values and for the multiscale DME. We have added text to the Results section as follows (L374-381):*

*“We evaluated the sensitivity of hierarchical clustering results to the choice of specific threshold (Supplementary Fig. 7a-e). As for the analysis of results across frequency band, groups of ROIs identified as clusters with a threshold of 33% in Figure 4 tended to cluster together for other threshold values, although their relative position in the one-dimensional representation of the dendrogram tended to vary. Results obtained using the multiscale DME approach were nearly identical to those obtained with t = 1 (Supplementary Fig. 7f), indicating that clustering was robust to the choice of the diffusion parameter t as well, consistent with results for inter-ROI distances above.”*

(6) As discussed thoughtfully in a recent review co-written by more than fifteen iEEG research centers (Mercier et al., 2022), there is no consensus on a given method to perform statistical analysis in the field of iEEG. For instance in the present study, the amount of data differ between participants both in term of data length and in term of contribution to the ROI/parcel (e.g., some result might be driven by a handful of participants that are more represented than the other for a given ROI). Also, it is recommended to provide some estimate of the representativeness or the result or at least to be explicit when 'random-effect' vs. 'fixed-effect' analysis was conducted.

*We agree with the Reviewer that there is no clear consensus on how to perform statistical analysis in the field of iEEG. A strong case can be made that there is no such consensus in most areas of neuroscience. 'Fixed-effects' and 'random-effects' are terms from regression analysis; however, we did not use regression methods for analyzing results across participants. Instead, we provide bootstrap estimates of the uncertainty of position in embedding space (for example, Figure 3) and permutation tests for the various inferential tests in the manuscript.* *Because bootstrapping and permutation tests are done at the participant level, these estimates are most comparable to a model accounting for random effects of participant. That is, they represent estimates accounting for participant level variation in the sample (rather than recording site-level estimates, with or without correcting for correlations within levels)*. *We do not feel it is correct to label these as "random" or "mixed" effects approaches. We cite relevant previous papers in the manuscript (L1126, 1130) that support the approaches used. We have added the following statement to Methods to clarify this point (L1138-1142):*

*“As the bootstrapping procedure was applied at the participant level, the variance estimates are most comparable to those of a model accounting for random effects of participant. That is, they represent estimates accounting for participant-level variation in the sample rather than recording site-level estimates, with or without correcting for correlations within levels.”*

(7) Most of the study is data driven except the original parcellation defining the ROI. A full data driven approach would have been appreciated. Could the authors discuss the potential influence of the first pooling step?

*We thank the Reviewer for raising this important issue. As we note in the manuscript (L215-216), the number and location of implanted electrodes was dictated solely by clinical considerations, and thus varied considerably between participants. Thus, recording sites were at discrete points and unique locations in each participant. We have clarified in the paper (L216-218) that assignment of recording sites to a finite number of pre-defined ROIs was a necessary step to combine data from multiple participants. We have also added the following statement to the Discussion acknowledging this limitation (L794-802):*

*“Finally, we note that while most of the analyses presented here were data-driven, the initial step of assignment of recording sites to specific ROIs was not. Unlike non-invasive neuroimaging methods such as fMRI, intracranial electrode recording sites sample activity discontinuously and at discrete locations. In addition, in order to pool data across participants, we were constrained by variable electrode coverage across participants. Thus, we were unable to consider structure at the finest scales due to these limits in sampling. Instead, we investigated higher levels of organization of brain regions. Our results provide insights into the relationship between network structure and function that can inform future studies with more uniform sampling.”*

Minors:
- Blurb: The last sentence could briefly summarize the results rather than suggesting some future applications.

*We appreciate this suggestion. We have revised the text as follows to incorporate the Reviewer’s suggestion while adhering to the Journal guidelines on the Blurb format (L56-60):*

*“We describe the organization of human neocortex on multiple spatial scales based on resting state intracranial electrophysiology. We provide evidence for hierarchical organization of auditory cortical regions and a limbic stream that parallels the classically described ventral and dorsal auditory processing streams, and identify network hubs in anterior temporal and cingulate cortex.”*

- In the introduction, the sentence related to sensitivity and specificity could be completed with the response made to the previous round of review.

*We appreciate this suggestion. We illustrate the relevance of sensitivity and specificity to functional connectivity patterns in L105-117 of Introduction. We hesitate to add more text because of the formatting constraints of the Journal.*

- Can the authors clarify the difference between the terms 'polysensory' and 'transmodal'?

*We have removed the words ‘polysensory’ and ‘transmodal’ from the paper for clarity.*

- Line 167: Please provide the metric used to estimate the variance (MAD would be more straightforward to understand than stddev).

*Thank you for pointing that out. We have clarified the metric as “standard deviation” in the manuscript, as requested (L157).*

- Line 176: This brain parcellation seems to not be described in the materials and methods.

*The brain parcellation scheme is described in Methods (Lines 907-932).*

- Lines 186 and 191: The word 'diffusion' might be confusing for the non-specialist as it is mostly used in reference to DTI. Please clarify briefly.

*We have clarified the meaning of ‘diffusion’ as requested. (L177-178).*

- Lines 192-193: Please add few words to explain how the SNR was computed.

*The meaning of SNR has been clarified as requested (Lines 184-185).*

- Figure 1: Maybe the electrodes outside the brain are not needed are they were not analyzed. For the depth (sEEG) electrodes would it be possible to use a depiction that allow their identification? (e.g., transparency or an outline).

*Thank you for bringing up this important issue. All recording sites depicted in Fig 1 were within cortical gray matter, as defined by analysis of each participant’s anatomical reconstruction data (as opposed to projection onto the common brain space). The reason some appear outside the brain in Figure 1 is due to the co-registration error that is inevitable given the considerable variability in individual brain anatomy. With that in mind, the template brain is shown for spatial reference purposes only. We have added a clarification to the caption of Figure 1 (L196-198):*

*“All depicted sites were within cortical gray matter; some appear as outside the brain due to individual variability in brain anatomy relative to the template brain.”*

*Regarding depth electrode contacts, the majority of these are already depicted in Figure 1 in the views most appropriate for a given ROI (as described in the figure caption). Locations of depth electrode contacts are not shown only for a minority of ROIs (frontal and parietal operculum, amygdala, hippocampus, putamen, globus pallidus, caudate nucleus, substantia innominate, and ventral striatum). These are denoted by asterisks in Fig 1c. Given the complexity and size of Figure 1, we prefer not to add additional panels to depict electrode coverage of these (mostly) deep brain structures.*

- Figure 3: Which estimate of variance was computed? (i.e., stddev, var or another metric).

*The variance estimates have been clarified in the caption of Figure 3 (L269-270): “The semiaxis lengths of each ellipsoid indicate the standard deviation of bootstrap estimates in each dimension.”*

- Lines 389-391: Please add a sentence to explain how a specific threshold can be chosen depending on the hypothesis testing.

*Thank you for this suggestion. We have added an example to Results (L403-404):*

*“For example, smaller sample sizes constrain hypothesis testing to a smaller number of brain subdivisions.”*

- Figure 4: Panel c is not present in the figure, only in the captions.

*Thank you for pointing out this error. We have corrected the figure caption.*

- Line 453: The term 'information' flow is misleading as not information metric was used.

*Text has been modified as requested (L447-449):*

*“These results are consistent with network organization depending on temporal scale, and are consistent with previous reports showing that mesial cortical structures regulate information flow on slower time scales [10].”*

- Lines 488, 502 and 511: Only Beta band is mentioned while other frequency bands were analyzed.

*We focus on gamma and beta bands as they are postulated to carry feedforward and feedback information, respectively, and thus would be expected to exhibit maximal band-specific differences. We have clarified this by revising text in the Introduction (L143-146):*

*“We applied exploratory statistical analyses to other beta band connectivity, reasoning that if gamma and beta band carry feedforward and feedback information, respectively, we expect the largest differences between these two bands. We present key findings from all bands to explore the sensitivity of our results to the choice of specific band.”*

- Line 547: Please provide the number of electrodes used here.

*We have added the number of recording sites (= 1591) at the indicated location in Results (L524). These data already appear in Supplementary Table 2.*

- Line 560: What was the rationale to use the SY parcellation rather than the parcellation used in Figure 1?

*The Schaefer-Yeo parcellation is based on functional connectivity in fMRI, which motivated its use in our fMRI-based dataset. The motivation for the parcellation used for iEEG data is that its resolution is best suited for keeping an adequate number of channels and participants within each ROI for analysis. We have added the following clarification (L535-538):*

*“We used a standard parcellation scheme developed for fMRI data (Schaefer-Yeo 400 ROIs; [88]) rather than the iEEG parcellation scheme introduced in Figure 1, as the latter scheme does not cover the entire brain and cannot be applied to automatically parcellate the fMRI data on a voxel-by-voxel basis.”*

- Figure 8: The scaling could be the same for the x axis and the y axis to show that the correlation is above the diagonal.

*The spatial scale of the data in embedding space is determined by the number of included ROIs, which is different between the partial fMRI and full fMRI measurements. Thus, the relationship between the distribution of the data and the diagonal is not considered. What is important, though, is the correlation between the two datasets, which is examined in Fig 8b.*

- Line 765: Why the 'high mean connectivity' was deleted?

*We felt that this text was redundant, as we show in Figure 5b that central location is highly correlated with mean connectivity.*

- Line 946: Due to the non-linear mapping between an average template and an individual brain, a linear coregistration is not recommended (see Mercier et al., 2022). Can the authors double check this processing step?

*The MNI coregistration was verified with a visual comparison of the transformed T1 with the template brain. In our experience, the difference between linear and nonlinear coordinates is typically small enough (<5 mm) to be negligible for the purpose of the ROI-based analysis. Importantly, the MNI coordinates were only used for plotting the recording sites from multiple participants to illustrate the overall extent of electrode coverage. MNI coordinates were not used for assigning sites to ROIs, nor were they used in any analyses presented in the study. We have added text to the Methods section clarifying this point (L902-906):*

*“The MNI coregistration was verified with a visual comparison of the transformed T1 with the template brain. Importantly, the MNI coordinates were only used for plotting the recording sites from multiple participants (Figure 1) to illustrate the overall extent of electrode coverage. MNI coordinates were not used for assigning sites to ROIs, nor were they used in any analyses presented in the study.”*

- Line 977: The classification of white/gray matter electrodes could be informed by brain segmentation provided by freesurfer. It is a pity that this processing step was not automatized.

*We appreciate the Reviewer’s suggestion, and we do have the option to use Freesurfer aseg white/gray parcellation as part of our anatomical reconstruction pipeline. In our experience, however, visual inspection of structural MRI reconstruction data is more reliable than any automated parcellation scheme.*

- Line 1021: Can the authors provide information about the spatial filter applied to 're-reference' the data?

*We apologize for not providing more detail about this aspect of the data preprocessing. We have revised its description in Methods (L981-994):*

- Line 1046: This sentence is repeating the one several lines above.

*Thank you for pointing this out. We have revised the text to eliminate this repetition.*

- Lines 1205-1224: Did the target sounds were included in the analysis?

*Both non-target and target trials were included in the analysis to improve the SNR of the recordings and maximize the sensitivity of the analysis. We have added the following clarification (L1192-1193): “Both non-target and target trials were included in the analysis to maximize its sensitivity.”*

**Reviewer #2**

Reviewer #2: Thanks to the authors for such a compelling rebuttal and according revision.

I can follow the authors on a vast majority of points raised, and I consider the revised ms. sufficiently transparent and clear for readers to make up their own mind on a few remaining minor issues of contention.

I look forward to these data being made publically available under a prominent open-source umbrella such as OSF.

*We thank the reviewer for their insightful comments and kind words. We agree that making these data available to the neuroscience community is important, and we are happy to do that. The University of Iowa IRB protocol under which these data were collected does not allow for making the raw data publicly available. We are able to share the data with interested researchers upon request and establishment of a data sharing agreement, which our institution agrees would be acceptable under the protocol.*
